# Long-term outcomes of flap reconstruction for hardware exposure in low-energy distal tibia fractures

**DOI:** 10.1016/j.jpra.2025.06.016

**Published:** 2025-06-28

**Authors:** Eleni Karagergou, Dimitrios Kitridis, Ektoras Kessidis, Nikolaos Platon Sachinis, Alexandros Givissis, Panagiotis Givissis

**Affiliations:** a 1st Orthopaedic Department, School of Medicine, Aristotle University of Thessaloniki, G. Papanikolaou Hospital, G. Papanikolaou Avenue, Thessaloniki, Greece; b 1st Orthopaedic Department, 424 Army General Training Hospital, Periferiaki street Nea Efkarpia, Thessaloniki, Greece; c School of Medicine, European University of Cyprus, Diogenous 6, Nicosia, Cyprus

**Keywords:** Tibial fractures, Ankle fractures, Wound dehiscence, Perforator flap, Ilizarov technique

## Abstract

Wound dehiscence and hardware exposure are significant complications following open reduction and internal fixation (ORIF) of low energy distal tibia and ankle fractures. This study aims to evaluate the long-term outcomes after removal of the exposed implants, application of an Ilizarov framework when needed and flap reconstruction.

A prospective case series study was conducted, including eight patients with wound breakdown and exposed hardware following ORIF for closed distal tibia and ankle fractures since January 2022. Data collection consisted of patients’ demographics, characteristics of the initial injury, wound microbiology and subsequent management. Patients were treated with removal of exposed implants, application of Ilizarov framework in cases of nonunion and flap reconstruction. Outcomes were assessed at 2 years postoperatively. The primary outcome measure was the percentage of clinical and radiological bony union, while secondary outcomes included wound complications, chronic osteomyelitis and patient-reported satisfaction using the LIMB-Q™ questionnaire.

The mean time from ORIF to removal of metal work was 8.3 weeks. Ilizarov framework was placed in five patients due to nonunion at the fracture site. A local propeller flap was selected and satisfactory wound coverage was achieved in all cases. The rate of bony union was 75 % at 2 years of follow-up. One patient developed chronic osteomyelitis and another one refractured his tibia. In terms of patient satisfaction, the appearance of the leg rated low, followed by function and patients’ expectations.

Once hardware exposure following ORIF for closed low energy distal tibia and ankle fracture is encountered, local flap reconstruction and targeted antibiotic therapy are essential for eradication of infection and promotion of bony union. Removal of hardware and replacement with an external fixation system are mandatory for late infections, although it should be re-considered in earlier infections, where hardware maintenance and suppression of infection may be equally effective as an alternative treatment.

## Introduction

Fractures of the distal tibia and ankle are difficult injuries to treat due to complexity of the various fracture patterns in conjunction with the thin skin and soft tissue envelope in this anatomical area. Following high or low energy injuries, these fractures are often complicated by skin necrosis and dehiscence due to the limited subcutaneous layer and absence of skin elasticity.^1^ Additionally, compartment syndrome can occur in both closed and open tibia fractures more often in high energy injuries where the soft tissues are at great risk.

Open reduction and internal fixation (ORIF) of distal tibia and pilon fractures provide an anatomic reduction but the percentage of wound complications has been reported as high as 37 %.^2^^,^^3^ In a large case series of 145 fractures of the ankle joint that involved the tibial plafond, 11 % and 6 % incidence of skin problems and osteomyelitis were reported, respectively, and three patients were subjected to limb amputation.^3^

According to the definition of fracture-related infection (FRI), wound breakdown following osteosynthesis of a fracture is considered a confirmatory criterion for the presence of infection and as such these injuries have to be managed.^4^ In cases of infection after fracture fixation (IAFF), the goal of treatment is healing of the fracture and avoidance of chronic osteomyelitis.^5^ In patients with early exposure of hardware, within the first 2 weeks, a biofilm forms but it may still be in an “immature” phase.^5^ If exposure occurs within the first 2–4 weeks and reduction is acceptable with a stable implant and adequate skin coverage can be achieved, retention of the metal work with debridement and antibiotics could be adequate for bony union.^5^^,^^6^

In delayed or late hardware exposure, production of the protective matrix called biofilm by the microorganisms around the implant provide a mechanical barrier to the host’s immune cells and antimicrobial therapies.^7^ Therefore, a staged approach with removal of the metal work and placement of an external fixation system is advised first, followed by systematic antibiotics and re-osteosynthesis at a second stage.^5^

When the skin is insufficient or necrotic, reconstruction with a local or free flap is mandatory to cover the fracture site and provide sufficient vascularity to manage infection and promote fracture healing. Although local flap options in high energy distal tibia fractures are diminished due to damage of the local perforator vessels, these flaps can be successfully used in low energy fractures following falls from small heights and torsion injuries around the ankle. In this study, we describe a series of patients with low energy distal tibia and ankle fractures who initially had ORIF and subsequently developed skin necrosis and wound breakdown with exposed hardware. The study aims to evaluate the long-term outcomes after removal of the exposed implants, application of an Ilizarov framework when needed and local flap reconstruction.

## Patients and methods

We conducted a prospective case series study with data collected since January 2022. Formal approval by the institutional review board was obtained and all patients provided their written informed consent. The study adhered to the STROBE guidelines. Our case series included patients with wound breakdown and exposed hardware after ORIF for closed distal tibia and ankle fractures that necessitated removal of metal work and flap coverage. All patients sustained low energy injuries subsequent to falls from small heights in combination with torsion around the ankle joint. These patients were referred to our tertiary major trauma center from peripheral district hospitals. Postoperatively, a minimum of 2 years follow-up was mandatory for inclusion in this study. Patients with shorter follow-up time, high energy injuries and open fractures as well as different primary methods of fixation—other than ORIF—were excluded from the study.

Data collection included demographics, type of fracture, time from injury to primary ORIF, time from ORIF to removal of metal work, replacement or not with an external fixation system, type of flap used for reconstruction, wound microbiology and duration of antibiotic therapy. The percentage of patients with clinically and radiologically united fractures and suppression of infection at 2 years follow-up was set as the primary outcome measure. This was equivalent to the percentage of patients with clinical cure. Secondary outcomes included short- and long-term complications, such as wound healing problems with the flaps and donor sites and presence of chronic osteomyelitis. Patient satisfaction was evaluated with the LIMB-Q™ questionnaire at the final follow-up.^8^ All participants filled out the scales concerning appearance, function, symptoms, expectations, financial impact, life impact, psychological and work, with a final score from 0 (worst) to 100 (best). A licensed agreement to use the LIMB-Q was obtained from McMaster University. Descriptive statistics were calculated using the Statistical Package for the Social Sciences (SPSS, IBM) software version 24.

## Results

**Figure 1:**
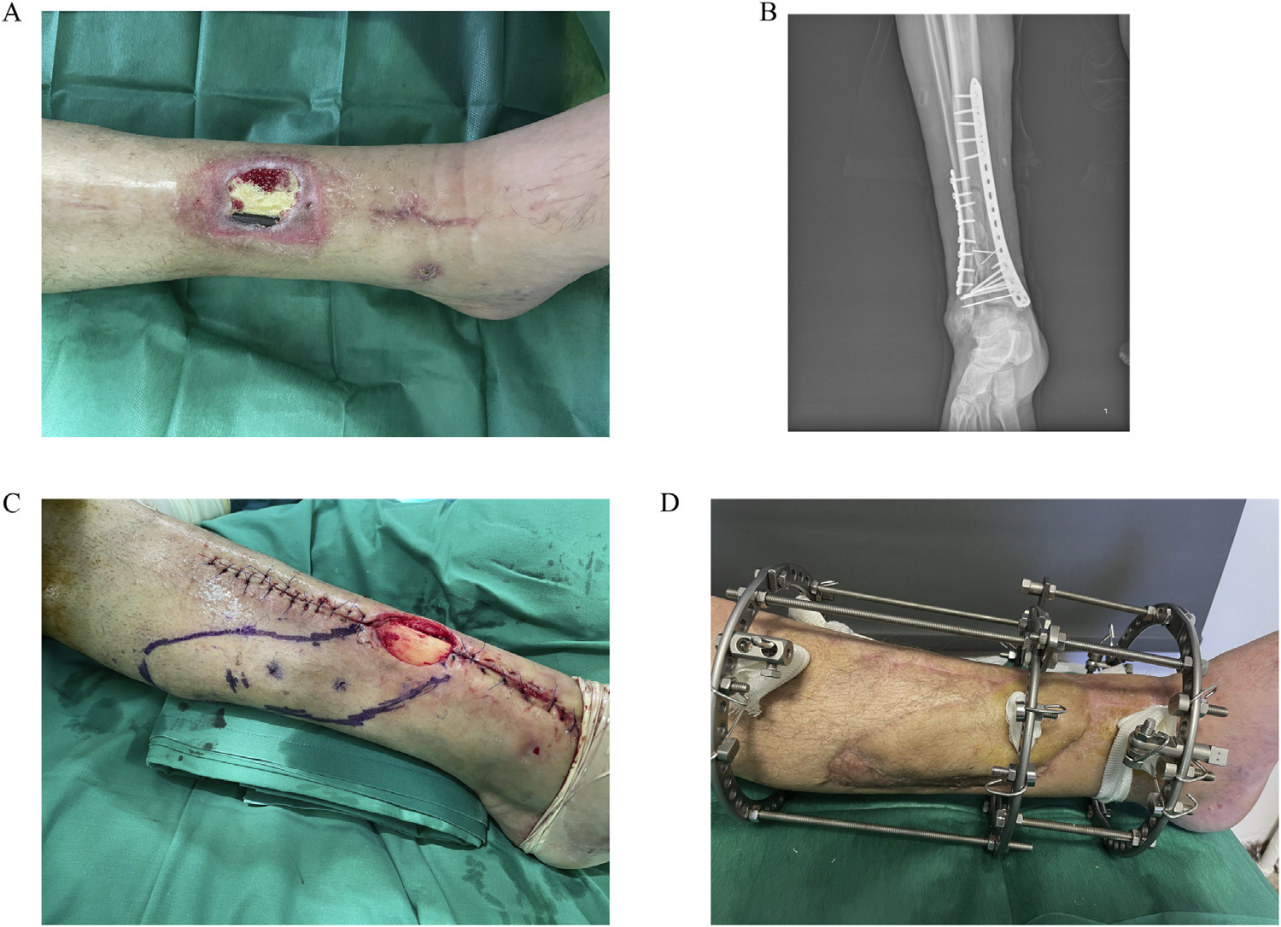
(A) A 74 year-old patient with wound dehiscence and implant exposure over the distal third of his anterior tibia. (B) X-ray of intra-articular distal tibia and fibula fracture stabilized with plates and screws. (C) Full-thickness defect with tibia exposure following surgical debridement and removal of hardware. Design of a local propeller flap to cover the defect. (D) Satisfactory wound healing after flap reconstruction and application of Ilizarov framework.

**Figure 2:**
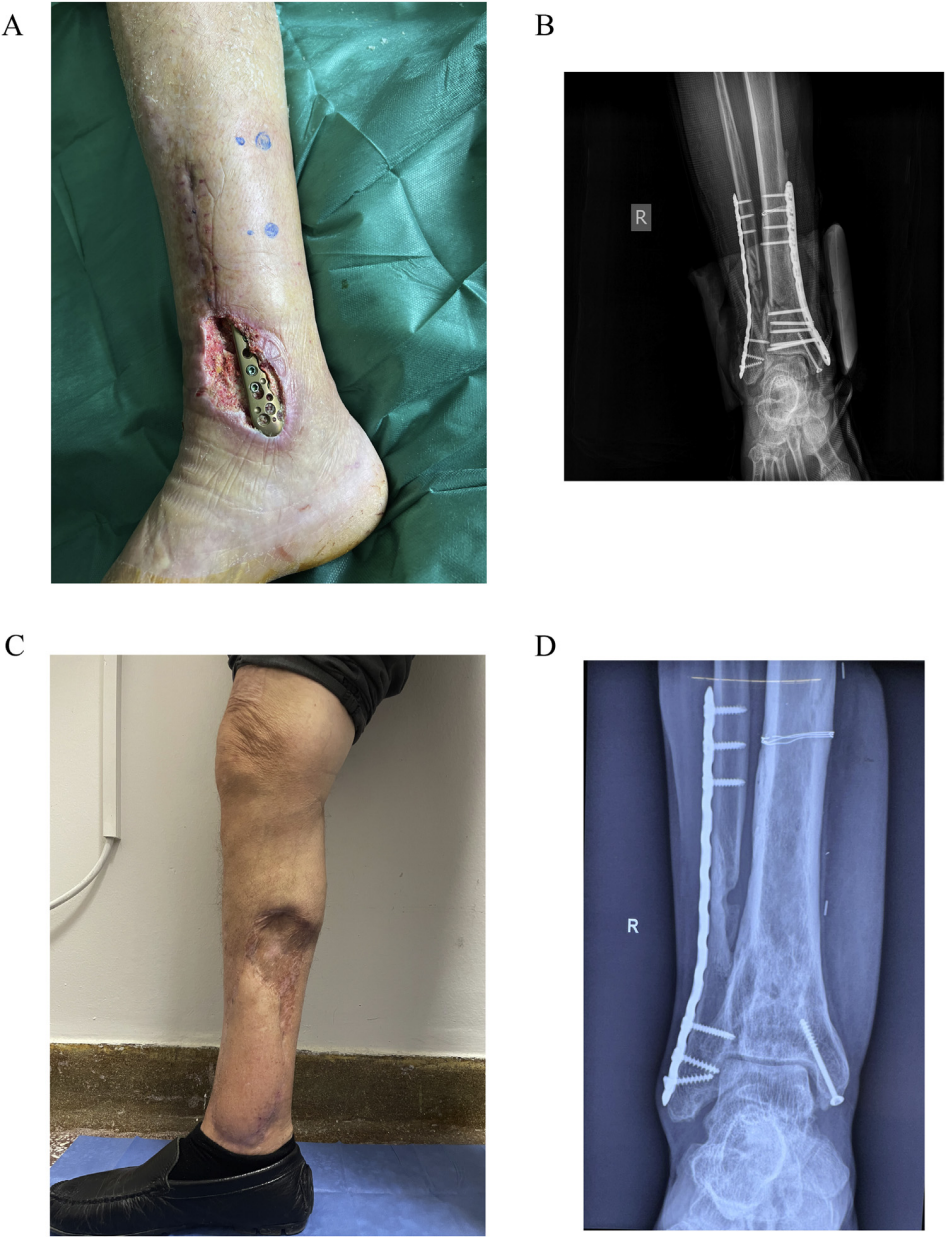
(A) A 72 year-old patient with implant exposure following ORIF of a comminuted distal tibia and fibula fracture. (B) X-ray showing the primary osteosynthesis with plates and screws. (C and D) Postoperative appearance of the reconstructed leg and x-ray showing bony union at 2 years of follow-up.

There were 4 men and 4 women with a mean age of 63.2 ± 11.02 years and a mean BMI of 26 ± 2.8. The fracture was at distal tibia diaphysis (*n* = 4), ankle (*n* = 3) and distal tibia/ankle (*n* = 1). The mean time from injury to primary ORIF was 1 ± 1.06 days, as reported both by the patient and the referral clinician. All patients had removal of the metal work at a mean time 8.3 ± 7.6 weeks since the primary ORIF and reconstruction with a local propeller flap based on perforator of posterior tibial (*n* = 7) or peroneal (*n* = 1) artery (Figure 1, Figure 2). The flap donor sites were covered with split-thickness skin grafts harvested from the thigh. Due to fracture non-union at the time of metal work removal, replacement with an Ilizarov framework system was selected in five patients. Two patients with solid bony union did not necessitate additional stabilization while one patient with a trimalleolar fracture and severe underlying infection, who appeared with a non-union over the medial malleolus required additional stabilization with two Kirshner wires after removal of the hardware. Wound microbiology is shown in Table 1. Apart from two patients with negative tissue cultures, the rest had microbial infections that necessitated a 6-week course of antibiotics.

**Table 1:** Patients’ demographics and characteristics of distal tibia and ankle fractures.

	Age	Gender	BMI	Type of fracture	Time from injury to ORIF	Time from ORIF to removal of metal work	Replacement with ex-fix	Wound microbiology	2-year bony union
1	73	F	22	Comminuted distal tibia/fibula	2 days	2.5 weeks	Yes	Capitis staphylococcus	Yes
2	43	M	28	Bimalleolar and distal tibia/fibula	2 days	3 weeks	Yes	Negative	Yes
3	54	F	23	Bimalleolar	2 days	24 weeks	No	MRSA	Yes
4	74	M	27	Intra-articular distal tibia/fibula	0 day	12 weeks	Yes	Acinetobacter - Stenotrophomonas	No
5	72	M	26	Comminuted distal tibia/fibula	0 day	4 weeks	Yes	Staphylococcus haemolyticus	Yes
6	70	F	31	Trimalleolar	0 day	5 weeks	No	MRSA Klebsiella Pseudomonas	No
7	61	F	26	Bimalleolar	2 days	13 weeks	No	Negative	Yes
8	59	M	25	Distal tibia/fibula	0 days	3 weeks	Yes	Staphylococcus haemoliticus	Yes

One patient developed flap congestion which was managed with leeches for 5 days, and subsequently resolved. Another patient had delayed wound healing over the skin graft donor site which finally healed after 10 weeks. In total, six out of eight patients presented with clinical and radiological bony union at 2 years of follow-up. This was equivalent to a 75 % cure rate. One patient returned with a sinus over the medial malleolus, draining pus, 6 months postoperatively. She had initially sustained a trimalleolar fracture with subsequent wound breakdown and polymicrobial infection and had a poorly controlled diabetes mellitus. The sinus and the underlying osteomyelitic bone were excised. Despite the long course of intra-venous antibiotics, she has developed non-union and chronic osteomyelitis for which she has been on low dose of oral trimethoprim/sulfamethoxazole to keep the infection suppressed. Another patient with a pilon fracture, had a fall 7 months postoperatively and refractured the distal tibia. He denied any further surgical treatment and up to now, he has developed nonunion.

**Figure 3:**
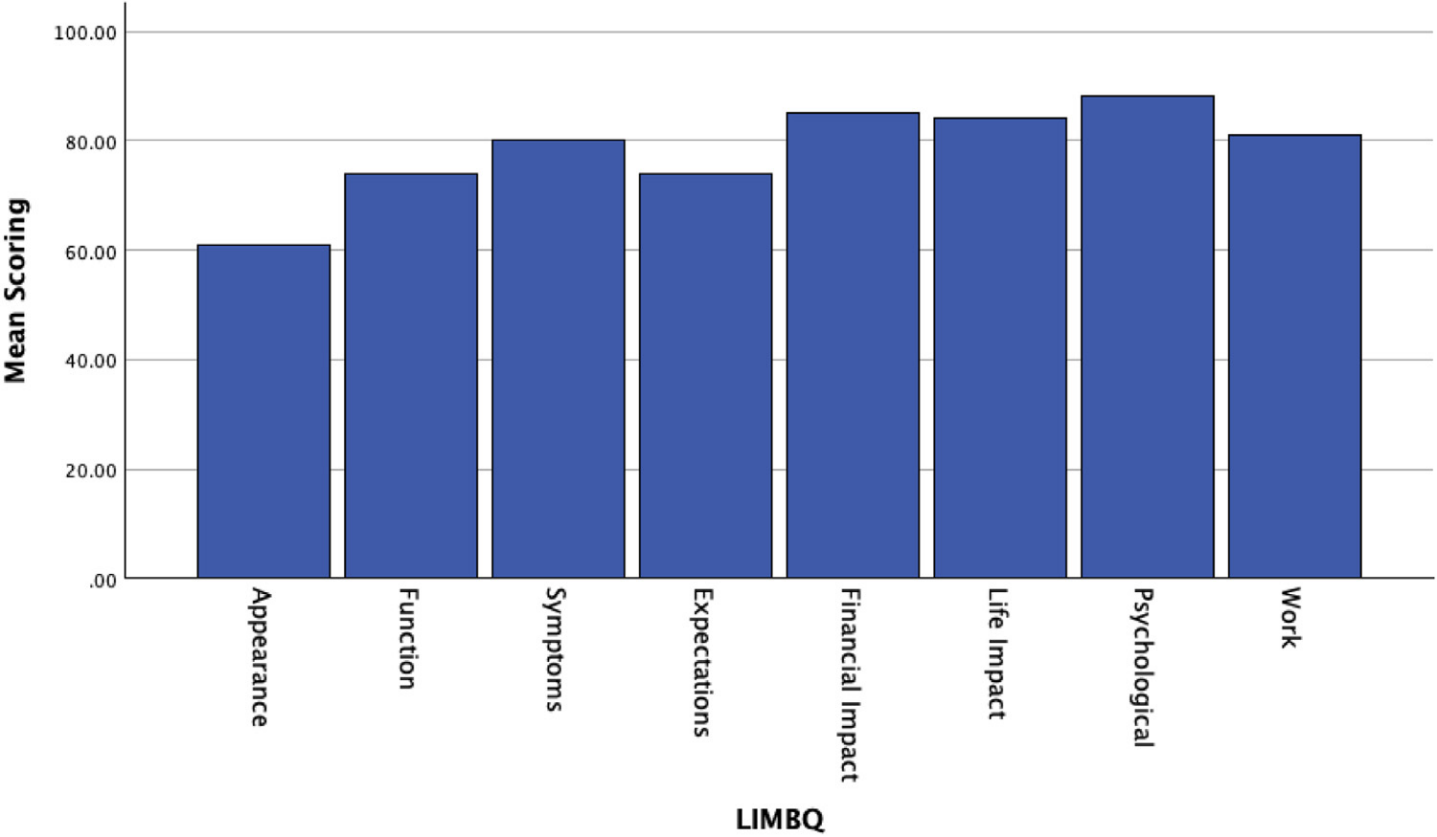
Mean scoring of LIMB-Q™ subscales.

The mean scorings of the LIMB-Q™ subscales concerning appearance, function, symptoms, expectations, financial impact, life impact, psychological and work are shown in Figure 3. The appearance of the reconstructed leg was rated low (61 ± 21), followed by function and patients’ expectations (73 ± 22 and 74 ± 15, respectively). All other subscales rated higher than 80.

## Discussion

Open reduction and internal fixation of closed or open distal tibia fractures is the gold standard to achieve stable and anatomic fixation, especially in cases with intra-articular extension.^9^ In low energy injuries, this principle usually results in good-to-excellent outcomes.^10^ In the elderly population though, these injuries are affected by osteoporosis and medical comorbidities (eg diabetes and vascular disorders) that may compromise the skin and soft tissue envelope. Complications such as skin necrosis, wound breakdown and osteomyelitis resulting in amputation were reported following ORIF of closed distal tibia or open pilon fractures with compromised skin.^10^ In a large series of fractures of the tibial plafond, Ovadia and Beals^3^ reported 11 % and 6 % incidence of skin problems and osteomyelitis, respectively, and three amputations. In another series, McFerran et al. noted 24 % and 17 % rate of wound breakdown and infection, respectively.^11^ In order to reduce the problems with soft tissue and bone healing in this difficult anatomical area, the timing and method of surgical management are very important to consider.

During the period of post-traumatic soft tissue swelling, the loss of skin elasticity renders primary closure risky and the wound is susceptible to wound breakdown. Therefore, ORIF was recommended either immediately (within 24 h) and prior to soft tissue swelling, or by delaying it and allowing its resolution.^12^ In our case series, half of the patients were primarily managed surgically during the second post-injury day, when the post-traumatic swelling could have rendered primary wound closure risky due to tension. To overcome this limitation, a staged approach was introduced to allow recovery of the soft tissues before definitive fixation.^12^^,^ ^13^ During the first stage, plating of the fibula and spanning external fixation were selected, followed by exchange of the external to internal fixation within 7–14 days. In this way, a decreased incidence of deep infection was reported.^12^

Apart from the timing of surgical management, the surgeon’s experience and skills play an equally important role to avoid postoperative complications. A poor surgical technique and its subsequent iatrogenic trauma can compromise vascularity to the skin, resulting in skin necrosis and wound breakdown in a difficult anatomical area with no skin elasticity. We strongly recommend to handle the skin and the soft tissues with respect and to avoid extension of skin incisions in areas where local perforator vessels are present, as these vessels may offer local reconstructive options if needed.

All patients in our series were good candidates for a local fasciocutaneous flap, as they sustained low energy injuries with absence of skin degloving and preservation of local perforators coming from the posterior tibial and the peroneal artery, facilitating the use of propeller-type flaps. These flaps have the advantage of replacing “like-with-like,” preserve the main vessels and reduce operating time when compared to free flaps.^14^ However, they were criticized due to concerning rates of partial and total flap necrosis.^15^ In a previous meta-analysis comparing free versus perforator propeller flaps in lower extremity reconstruction, the failure and overall complication rates were comparable.^14^ In our study, we had no partial or total flap failure and all patients had successful coverage of the defect. Adequate release of the perforator is crucial to avoid twisting or kinking after rotation. Only one flap showed venous congestion, which subsided after leech therapy for 5 days.

An important clinical dilemma is whether the exposed metal hardware should be left in place or removed. In some fractures, removal of hardware before bony union can be detrimental due to loss of adequate reduction while an external fixation system cannot always restore it. Bonnevialle^6^ suggests that in the event of successful fracture reduction and optimal mechanical performance of the fixation material, the hardware may be left in place if diagnosis and treatment start within 3–4 weeks. Wen et al. reported a series of seven patients who had local flap reconstruction to cover wound dehiscence over fracture of the distal tibia with exposed hardware, by keeping the metal work.^16^ They reported solid bony union in all patients, although only five out of seven could walk normally with no pain at a mean follow-up of 10.6 months. Occasionally, suppressive treatment of the infection has a role while bone healing is the primary objective. The internal fixation device can then be retrieved once consolidation is obtained.^17^ In a multi-center retrospective study of patients with early (within 6 weeks) postoperative infection following internal fixation of fractures in various anatomical areas, 121 participants had maintenance of hardware and antibiotic suppression until osseous union. The overall failure rate was 29 % as defined by nonunion and inability to clear the infection resulting in amputation and chronic infection.^17^

In our series, five patients had removal of the hardware and replacement with an Ilizarov framework system, which proved to be adequate for bone healing. One of these patients though refractured his tibia but declined further surgical treatment. This approach is certainly more aggressive when compared to hardware maintenance and suppressive antibiotics and increases morbidity and treatment duration. However, the mean time from ORIF and wound dehiscence to referral and removal of metal work was 8.3 weeks. Under these circumstances, hardware maintenance was not possible. There was a subgroup of four patients with early presentation, within 4 weeks, that we could have possibly kept the metal work and suppress infection with antibiotics. However this approach was not selected and instead, removal of hardware and replacement with the Ilizarov fixation system resulted in satisfactory bony union.

One of the main strengths of this study is the correlation between a specific treatment strategy for patients with exposed metalwork in distal tibia and its impact in patients’ life through the LIMB-Q™ patient reported outcome measure. Postoperative appearance after propeller flap reconstruction was rated low (61 ± 21) at 2 years of follow-up. Local fasciocutaneous flaps, despite offering a “like-to-like” salvage reconstruction, rated low due to the appearance of the skin grafted flap donor site and the oedema which does not subside in the long-term. Unfortunately, there is currently a lack of published studies to compare appearance of propeller flaps with other reconstructive methods in lower limb. Function and patients’ expectations were also rated low (73 ± 22 and 74 ± 15, respectively), reflecting the complexity of these injuries and the long-term morbidity that follows them. Therefore, avoidance of such complications by selecting the appropriate timing and method of initial treatment is of paramount importance.

Despite the prospective nature, this is a small case series of patients referred mainly by District Hospitals to our Centre. Wound breakdown and exposure of hardware after ORIF for closed low energy distal tibia and ankle fractures are not common and patient recruitment remains low. This study demonstrates that local propeller flap reconstruction is a viable option for managing hardware exposure in low-energy distal tibia fractures, achieving a 75 % bony union rate at 2 years. While previous studies have debated hardware retention versus removal, our findings suggest that removal with external fixation is an effective option even in early-stage infections (<4 weeks), where hardware maintenance with antibiotic suppression remains a potential alternative. The limitations of this study include the small sample size and lack of a comparative control group.

The design of a randomized controlled trial to study the outcomes of maintaining versus removing the hardware, before flap reconstruction, necessitates the participation of multiple centers, as these cases are rare. Therefore, these prospective trials are strongly recommended but difficult to achieve.

In conclusion, wound dehiscence and hardware exposure following ORIF for closed low energy distal tibia and ankle fractures, are challenging cases to treat. The focus should be on prevention by selecting the appropriate time and method of treatment. Once this complication is encountered, local flap reconstruction and targeted antibiotic therapy are essential for eradication of infection and promotion of bony union. Removal of hardware and replacement with an external fixation system are mandatory for late infections. In early infections, within 3–4 weeks, maintenance of hardware and suppression of infection until bone consolidation might be another alternative method with lower morbidity and shorter treatment duration.

## Funding

None.

## Ethical approval

The study was approved by the scientific review board (363/17.5.2022).

## Declaration of competing interest

The authors declare that they have no known competing financial interests or personal relationships that could have appeared to influence the work reported in this paper.
